# Barriers and Enablers in Implementing Electronic Consultations in Primary Care: Scoping Review

**DOI:** 10.2196/19375

**Published:** 2020-11-12

**Authors:** Rebecca Baines, John Tredinnick-Rowe, Ray Jones, Arunangsu Chatterjee

**Affiliations:** 1 University of Plymouth Plymouth United Kingdom

**Keywords:** remote consultation, COVID-19, implementation science, primary health care, patient participation, referral and consultation

## Abstract

**Background:**

Often promoted as a way to address increasing demands, improve patient accessibility, and improve overall efficiency, electronic consultations are becoming increasingly common in primary care, particularly in light of the current COVID-19 pandemic. However, despite their increasing use, a theoretically informed understanding of the factors that support and inhibit their effective implementation is severely limited.

**Objective:**

With this scoping review, we sought to identify the factors that support and inhibit the implementation of electronic consultations in primary care.

**Methods:**

In total, 5 electronic databases (PubMed, Medline, Embase, CINAHL, and PsycINFO) were systematically searched for studies published in 2009-2019 that explored the impact and/or implementation of electronic consultations in primary care. Database searches were supplemented by reference list and grey literature searches. Data were analyzed using inductive thematic analysis and synthesized using Normalization Process Theory (NPT).

**Results:**

In total, 227 articles were initially identified and 13 were included in this review. The main factors found to hinder implementation included awareness and expectations; low levels of engagement; perceived suitability for all patient groups, conditions, and demographics; cost; and other contextual factors. Reports of information technology reliability and clinical workload duplication (as opposed to reduction) also appeared detrimental. Conversely, the development of protocols and guidance; patient and staff education; strategic marketing; and patient and public involvement were all identified as beneficial in facilitating electronic consultation implementation.

**Conclusions:**

This review highlights the need for proactive engagement with patients and staff to facilitate understanding and awareness, process optimization, and delivery of coherent training and education that maximizes impact and success. Although the necessity to use online methods during the COVID-19 pandemic may have accelerated awareness, concerns over workload duplication and inequality of access may remain. Future research should explore health inequalities in electronic consultations and their economic impacts from multiple perspectives (eg, patient, professional, and commissioner) to determine their potential value. Further work to identify the role of meaningful patient involvement in digital innovation, implementation, and evaluation is also required following the rapid digitization of health and social care.

## Introduction

With a 16% increase in the number of general practitioner (GP) consultations between 2007-2014 in England alone, primary care is considered to be at “saturation point” [1]. General practice is often described as facing increasing demand, reduced accessibility, and heightened patient dissatisfaction [2,3]. As a result, primary care providers are being increasingly encouraged to adopt alternative, more digitally focused methods of care provision [4], particularly in light of the recent COVID-19 outbreak. This drive toward digital platforms is reflected on an international scale [5-8], with digital platforms often promoted as a way to relieve pressures on existing services and improve accessibility, efficiency, and cost-effectiveness while simultaneously promoting self-management and patient-centered care [1,3,4,9]. However, critical exploration of these assumptions is severely limited.

Within the United Kingdom, prior to the COVID-19 pandemic, there were two main electronic consultation providers: askmyGP and eConsult, the latter of which was previously known as WebGP [10,11]. These systems are online triage tools designed to provide patients with an alternative way of contacting their GP practice [4]. For the purposes of this article, we define the term electronic consultations as an online service that enables patients to access advice and care from a primary care practitioner or staff member by combining preliminary health issues and symptom checking with appointment booking. These products are distinct from video-based consultations that a doctor might use, such as Babylon, AccuRx, or LIVI. In the United Kingdom, electronic consultations are also distinct from their use in North America as a video system to communicate between family doctors and specialists in hospitals.

While some research has explored the benefits of electronic consultations and the experiences of health care professionals using them, such work is often critiqued for its overreliance on pilot studies [3,4]. Other criticisms of existing literature also include a limited understanding of the cost implications for both health care professionals and patients [12-14], and a predominate focus on professional perspectives [1,15], with limited exploration of patient experiences and expectations [3,6,11]. Furthermore, of the limited research conducted, the majority of it has focused on the experience of using electronic consultations, as opposed to the practicalities of implementing such technology. Finally, in spite of their increasing use, a theoretically informed understanding of the factors that support or inhibit the implementation of electronic consultations is severely limited [4,5,8,11], particularly in a UK setting [16]. This review seeks to address this gap by identifying the factors that support and inhibit the implementation of electronic consultations in primary care using Normalization Process Theory (NPT) as a theoretical framework. This scoping review is well-timed given the rapidly increasing use of electronic consultations in light of the COVID-19 outbreak and the resulting mandatory shift toward total triage in primary care [4]. Although focused on general practice and electronic consultations, the implications of this review may be relevant to other digital forms of health and care technology and their subsequent implementation.

## Methods

### Design

A scoping review was conducted due to their ability to map existing evidence in an emerging field (such as electronic consultations), identify gaps in existing understanding, and incorporate different study designs that can be grouped together to evaluate a particular topic of interest [17,18].

### Search Strategy

As advised by an information specialist, 5 databases (PubMed, Medline, Embase, CINAHL, and PsycINFO) were systematically searched using the search terms “econsult” OR “electronic consultation” OR “WebGP” OR “non face-to-face consultations” OR “technology mediated consultations” AND “primary care” OR “GP” OR “general practice.” Search terms were designed and reviewed using the Peer Review of Electronic Search Strategies (PRESS) guidance [19].

A grey literature search was also conducted to ensure sufficient inclusivity and coverage. Grey literature was defined as “that which is produced on all levels of government, academics, business and industry in print and electronic formats, but which is not controlled by commercial publishers” [20]. The peer-reviewed search strategy was also used in Google Scholar.

### Inclusion and Exclusion Criteria

Research studies that explored the impact or implementation of electronic consultation platforms designed to be used by a primary care clinician and patient, published in the English language, of any study design (including opinion pieces and editorial letters) were included. Research studies that were not published in the English language or focused on electronic consultation platforms outside of primary care (eg, between non–primary care specialties) were excluded to retain a relevant focus of interest.

While the researchers aimed to be inclusive, due to limited resources, a sensitive translation of non-English texts could not be provided. Finally, to ensure only the most contemporary literature was included, a time limit was applied (January 1, 2009, to January 31, 2019). Literature searches were conducted on February 1, 2019.

### Screening and Eligibility

All identified articles were screened using a two-stage process. First, the title and abstract of all identified articles were reviewed independently by two researchers using the inclusion criteria outlined above. This process was facilitated using Rayyan, software for conducting reviews [21]. The full texts of potentially relevant articles were then reviewed for inclusion. Any disagreements were resolved through the inclusion of a third researcher. Database searches were also supplemented by reference list searches of included studies.

Data was extracted from the included studies independently by two reviewers using a pilot tested extraction form. Data extracted included author names, date of publication, setting, study type, sample, analysis method, and reported findings/author interpretations.

### Analysis

Included studies were initially coded independently by two reviewers using inductive thematic analysis as outlined by Braun and Clarke [22]. Identified themes were then synthesized using NPT as outlined below by the same two reviewers.

### Synthesis

Data was synthesized using NPT [23] as an analytical framework. NPT was originally developed to understand the embedding of new, particularly complex technologies in health care systems [23], providing a clear rationale for its inclusion in this research. As outlined by Murray [23], NPT is underpinned by four constructs that often operate simultaneously:

1. Coherence: how people make sense of a new technology/system/process.
2. Cognitive participation: how people engage with a new technology/system/process.
3. Collective action: how people make new technologies/systems/processes work in practice (or not).
4. Reflexive monitoring: how people assess the value of new technologies/systems/processes.

For the purposes of this article, we report findings on the first three domains of NPT (coherence, participation, and collective action). As previously mentioned, of the limited research previously conducted, most has focused on professional experiences (eg, reflexive monitoring). We therefore focus on the three remaining domains of NPT to avoid duplication.

### Quality Appraisal

In line with scoping review practice [18], included studies were not quality appraised.

### Ethical Approval

Participation in this research was entirely voluntary. All participants gave full informed consent. The University of Plymouth, Faculty of Health and Human Sciences (Reference number 18/19-1060) provided ethical approval.

## Results

### Overview

**Figure 1:**
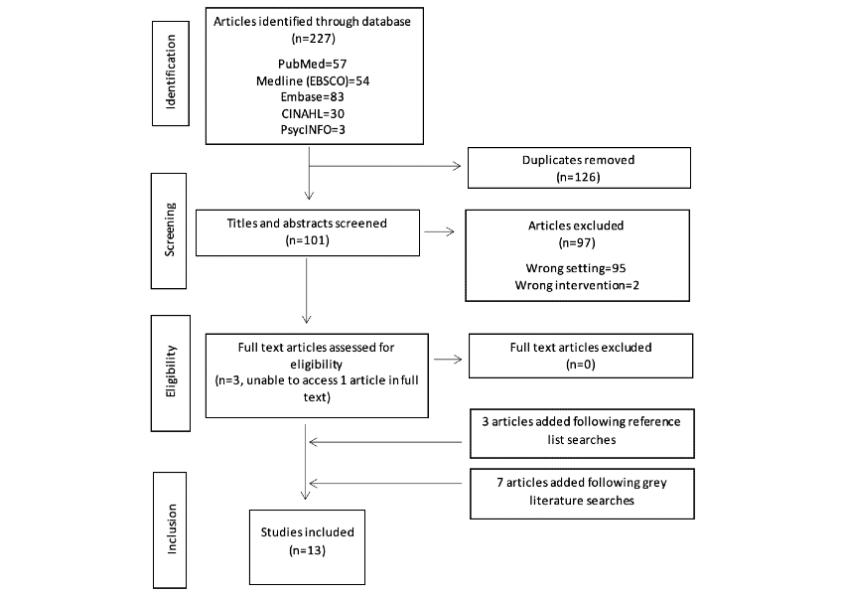
PRISMA diagram of included studies. PRISMA: Preferred Reporting Items for Systematic Reviews and Meta-analyses.

From the 227 articles initially identified, 13 were included for the purposes of this review (Figure 1). Table 1 summarizes the characteristics of the included studies.

**Table 1:** Characteristics of included studies.

Authors	Country	Study type	Intervention	Setting	Participants and databases	Analysis method
Atherton et al, 2018 [24]	England and Scotland	Mixed methods case design	Alternatives to face-to-face consultations	General practices with varied experience of implementing alternatives to face-to-face consultations	Patients and practice staff	Descriptive statistics, multivariate analysis, and coding of qualitative data from EMIS Health
Banks et al, 2018 [4]	West of England	Qualitative	eConsult	General practices that piloted an e-consultation system for 15 months during 2015 and 2016	23 semistructured interviews with staff members	Thematic analysis of interview data
Cowie et al, 2018 [25]	Scotland	Mixed methods	eConsult	11 general practices piloting eConsult	44 semistructured practice staff interviews, 1 focus group (4 staff), and 291 patient survey responses	Health economics, thematic analysis and coding, patient survey, descriptive statistics
Carter et al, 2018 [6]	South West England	Mixed methods	WebGP	6 GP practices in Devon	Six practices provided consultations data; 20 GPs completed case reports (regarding 61 e-consults); 81 patients completed questionnaires; 5 GPs and 5 administrators were interviewed	Statistical analysis of surveys, thematic analysis of interview data
Farr et al, 2018 [3]	South West England	Mixed methods	eConsult	6 general practices	23 practice staff interviews, patient survey data for 756 e-consultations from 36 practices	Economic analysis on usage and costs, Normalization Process Theory, inductive thematic analysis, patient survey and record data statistical analysis
Ogden, 2018 [26]	N/A^a^	Opinion piece	Online consulting	N/A	N/A	Descriptive statistics
Marshall et al, 2018 [8]	N/A	Opinion piece	Online consulting	N/A	N/A	N/A
Casey et al, 2017 [1]	N/A	Mixed methods case study	Online consultation system	Inner-city general practice	Information technology developers, clinicians, and administrative staff	Thematic and discourse analysis of interview data
Edwards et al, 2017 [16]	South West of England	Observational study	eConsult	South West of England	eConsult data obtained from 36 general practices	Economic analysis, website analytics, survey-based statistical analysis
Wise, 2017 [10]	N/A	Opinion piece	Online consultations	N/A	N/A	N/A
Hanna et al, 2012 [27]	Scotland	Qualitative	Non–face-to-face consultations	Scotland	20 semistructured interviews with general practitioners	Thematic analysis of interviews
Mair et al, 2012 [28]	N/A	Systematic review	eHealth systems (only information pertaining to online consultations recorded)	N/A	MEDLINE, EMBASE, CINAHL, PsycINFO, and the Cochrane Library were searched for reviews published between January 1, 1995, and March 17, 2009. Studies had to be systematic reviews, narrative reviews, qualitative meta-syntheses or meta-ethnographies of eHealth implementation	Evidence synthesis
Hanna et al, 2011 [15]	Scotland	Mixed methods	Non–face-to-face consultations	Scotland	600 practice manager questionnaire responses, 20 practice manager interviews	Chi-squared test from a survey, thematic analysis of interviews

^a^N/A: not applicable.

As demonstrated in Table 1, most of the research published in the last two years has used a mixed methods design. There has been a particular focus on the introduction of electronic consultations in South West England and Scotland. Our results are presented in line with the three selected domains of NPT as outlined above. We begin by presenting the factors that inhibit implementation, followed by those that facilitate implementation.

### Factors That Inhibit Electronic Consultation Implementation

#### Coherence: What Factors Inhibit the Understanding of Electronic Consultations?

##### Awareness and Expectations

A lack of understanding and awareness about the purpose of electronic consultation systems appears particularly problematic in their implementation and impact [24]. Patient understanding and awareness was reportedly mixed, with the effectiveness of electronic consultation advertising often called into question [16,25]. Patients were often not aware of alternative methods available to them, and how to access or operate such services [16,24]. Atherton suggests this is a matter for individual practices rather than policy directives [24].

A difference in staff and patient expectations regarding the appropriate use of electronic consultations was also reported [3]. This primarily related to the inappropriate use of electronic consultations to get a timelier face-to-face appointment as later discussed.

#### Cognitive Participation: What Factors Inhibit How People Engage With Electronic Consultations?

##### Low Uptake

Studies identified a low uptake as a further barrier to electronic consultation implementation [1,3,4,6,11,16,24]. Engagement levels were often reported to be much lower than expected [3]. For example, Banks et al [4] reported that only around 6% of practices used some form of electronic consultation. Similarly, Edwards et al [16] concluded that of the 36 practices reviewed, there was an average of 2 electronic consultations per 1000 patients per month. Such findings mirrored calculations by Cowie and colleagues [11]; the highest submission rate reported was almost 0.1 consultations per patient per year.

##### Suitability for Certain Patient Groups, Conditions, and Demographics

The suitability of electronic consultations for certain patient groups, conditions, and demographics was also called into question. Electronic consultations were considered more suitable for certain patient groups with discrete, “straightforward,” or familiar ongoing treatment queries [1,3,24,27]. In comparison, face-to-face consultations were reportedly preferred for new, acute, or complex cases that required physical examination and/or regular monitoring [3,4,16,24,26]. As a result, some health care professionals expressed concern that patient groups including those with long-term conditions, multimorbidity, and/or mental health problems would be disadvantaged or unintentionally excluded from electronic consultation opportunities [8,24]. Some GPs repeatedly expressed concerns that introducing a new technology could create or widen existing inequalities in access to health care [3,27,29]. Atherton acknowledged the purposeful selection of patients by health care professionals, including receptionists and administrators, based on their assumptions of who they felt would be able to use electronic consultations appropriately [24], highlighting further concerns regarding unintentional or intentional exclusion.

In regard to demographics, included articles also suggested that women are almost twice as likely to use electronic consultation systems than men [6,11,16]. Younger patients are also more likely to engage with electronic consultation systems, with levels of engagement typically declining with age [3,6,11,16,24]. Levels of education, language [3], and employment were also shown to influence electronic consultation use [6,24,27].

##### Contextual Factors: Practice Size, Deprivation, and Geographical Location

Following this, a range of contextual factors including practice size, deprivation, and geographical location appeared to influence electronic consultation implementation and effect [29]. Hanna et al [29] suggested that the bigger the practice size, the more support available, including a website, an information technology (IT) manager, and a triage team, thereby influencing implementation efforts and sustainability [29].

##### Cost

Finally, cost was reported as a significant barrier to electronic consultation engagement and implementation [3,4], with limited evidence available to justify its associated expense [4,11]. As suggested by Farr et al [3], costs often prohibited long-term engagement with electronic consultation systems.

#### Collective Action: What Factors Inhibit How People Work With Electronic Consultations?

##### Additional Time and Increased Workload

In regard to how people work with electronic consultations (collective action), the majority of included studies reported an increase in clinician workload. This was often attributed to additional follow-up and/or face-to-face consultations due to a lack of sufficient information being provided [1,4,6,11,16,27,28]. Although one study acknowledged a potential benefit of having the clinical issue documented prior to a face-to-face appointment [4], this conclusion was contradicted by another article that found that follow-up appointments were typically longer than the national average (14.5 minutes versus 9.2 minutes for face-to-face) [16]. However, it is important to note that this disparity may be a result of a lack of GP continuity between electronic and follow-up consultations [3].

GPs also reported significant difficulties in establishing a patient’s primary concern, following an inability to probe for further information [3,4,16]. Where reported, most electronic consultations resulted in either follow-up phone calls or face-to-face appointments, leading GPs to report a duplication in their workload, as opposed to a desired reduction [1,3,6]. The relatively low uptake of electronic consultation systems as previously described appeared to exacerbate this concern [11].

An increase in administrative workload was also reported, particularly the frequent need to contact patients regarding their electronic consultation outcome or to arrange a face-to-face or telephone appointment [11]. Cowie et al [11] reported that most practices made at least three attempts to contact patients, frequently reporting that contact could not be made. The use of a withheld number was suggested as a possible explanation. Some practices have developed methods to facilitate email contact as a solution to this problem, although issues of data security and protection remained a concern [11].

##### Medicolegal Concerns

Linked to concerns of data security and protection, medicolegal issues were also identified as a barrier to electronic consultation implementation and use. Concerns about the negotiation of clinical risk and diagnosis uncertainty were repeatedly expressed within the context of an increasingly litigious culture [8,27,29].

##### Potential to “Game” the System

As previously mentioned, some practice staff members expressed concern regarding “patients gaming the system” [4], with some reportedly using the system to achieve a timelier face-to-face appointment [3,4,6,11]. As reported by Banks et al [4] and others, staff often felt patients could get an appointment quicker using electronic consultation methods by circumventing traditional, often telephone-based appointment systems. The responsibility of the practice to contact the patient within a specified time frame, often by the end of the next working day [4,6], as well as the content of marketing material [11], appeared to influence this perception.

##### IT Reliability

Finally, the reliability of technology was seen as a potential barrier to electronic consultation implementation and use. As reported by Hanna et al [29], some interviewees felt new IT systems were highly reliable, while others expressed concern about network availability and speed [27,29]. The interoperability of IT systems was also identified as influential in electronic consultation implementation [3,11], with competing or conflicting IT systems proving to be problematic.

### Factors That Facilitate Implementation

In addition to the barriers outlined above, a number of facilitating factors were also identified in regard to electronic consultation implementation and use. Each are discussed in turn below.

#### Coherence: What Factors Facilitate the Understanding of Electronic Consultations?

##### Protocols and Strategies

First, the provision of protocols, strategies, and/or guidance including medicolegal advice was considered integral to the effective implementation of electronic consultations [11,26-28]. As identified by Cowie et al [11], the development of a clear strategy for introducing electronic consultations prior to any implementation was considered fundamental. Such protocols should be developed in collaboration with staff, patients, and electronic consultation champions (as discussed below) [11].

##### Education

Following this, quality patient and staff education on what electronic consultations are, what they are not, and when to use them was widely encouraged [3,6,11]. Cowie et al [11] recommended the construction of practice process diagrams along with clear objectives to facilitate staff understanding and awareness. An exploration of staff expectations was also considered integral [11].

##### Focused Marketing

Linked to the provision of education was a desire for more focused marketing [6,11,26]. Identified ways of promoting and marketing electronic consultations included leaflets, clear website positioning, posters, and recorded telephone messages [26]. As suggested by Ogden et al [26], recorded messages appeared particularly influential when recorded by a doctor.

##### Patient and Public Involvement and Wider Network Development

Combining the development of protocols, education, and marketing, patient and public involvement was also considered integral prior to and during any electronic consultation implementation to ensure acceptability and understanding [3,11]. Despite this, few practices reported engaging with patients in electronic consultation design, implementation, or evaluation [3]. This may explain some of the issues identified regarding patient understanding and awareness. Patient participation groups (PPGs) were identified as a beneficial resource for facilitating patient engagement, as were patient and public involvement workshops to provide feedback on electronic consultation systems, protocols, and experiences [11].

#### Cognitive Participation: What Factors Support How Stakeholders Engage With Electronic Consultation?

##### Staff Training

Moving on to cognitive participation, how stakeholders engaged with electronic consultations, adequate staff training that addressed how electronic consultations operate, how electronic consultations fit with existing practice processes, and individual responsibilities were considered essential for successful implementation [8,11,26-28].

##### eConsultation Champions

An electronic consultation champion was also considered helpful in ensuring effective implementation and engagement by promoting its use among patients and more reticent staff members [11,28]. However, the risk of jeopardizing staff and patient commitment by recruiting a critical champion was also acknowledged [28].

#### Collective Action: What Factors Facilitate How Stakeholders Work With Electronic Consultations?

##### Strategic Marketing

With regard to collective action (how stakeholders work with electronic consultations), strategic marketing or signposting was identified as a facilitating factor [3,6,11]. For example, signposting patients to use electronic consultations in certain situations where only remote GP access was likely to be required, including follow-up appointments, general administrative queries, repeat prescriptions, and general advice [11]. A more focused marketing strategy was considered to help improve overall efficiency, ensuring patients who might benefit from the service most were directly encouraged to engage with it [11].

##### Notification Alerts

In response to concerns of engaging in telephone “ping-pong,” a desire for the development of an alerting system to inform patients of an incoming call following an electronic consultation request was also expressed [6,11]. Some practices already provide an estimated time for patient call-backs, clearly informing the patient that this may be from an unavailable or unknown number [11]. The development and effective incorporation of email contact was also expressed as a desirable solution to improving electronic consultation implementation and effectiveness [11], although this is reliant on the smooth integration of electronic consultations into existing IT systems as described below [28].

##### Integration of Technology and Adequate Resourcing

Mair et al [28] suggested clinicians may be deterred or become resistant to working with a system that adds complexity or requires additional effort and time. IT support was therefore considered integral to implementation [27], as was adequate resourcing, particularly financial support [28].

##### Pairing of GPs With Patients

Finally, the possibility of allocating electronic consultations to GPs who had had previous contact with the patient was seen as an effective way to facilitate implementation [3]. Table 2 provides a visual representation of the barriers and facilitators outlined in this review, according to the relevant domains of NPT.

**Table 2:** Identified barriers and facilitators to electronic consultation implementation and use.

Normalization Process Theory domain	Barriers	Facilitators
Coherence	Lack of understanding regarding its purpose and intended use	Development of protocols, strategies, and guidance, including medicolegal advice Patient and staff education Focused marketing Wider consultation with patients and staff members prior to implementation
Participation	Low uptake Mainly administrative requests Suitability for certain patient groups and conditions Contextual factors including practice size, deprivation, and geographical location Cost Limited patient involvement	Staff training Strategic/targeted patient use for those most likely to benefit from electronic consultations Electronic consultation champion
Action	Purposeful patient selection. Additional time or increased workload Medicolegal concerns The potential to “game” the system Information technology reliability	Effective signposting informing patients of when to use electronic consultation and when not Notification alerts to alleviate administrative issues related to contacting patients Integration of technology and adequate resourcing Pairing of general practitioner with prior patient contact

## Discussion

### Summary

This review addressed an identified gap in existing literature by developing a theoretically informed understanding of the factors that support and inhibit the practical implementation of electronic consultations in primary care [4,5,8,11,16]. Review findings suggest limited staff and patient awareness and understandings; low levels of engagement; perceived suitability for all patient groups, conditions, and demographics; cost; and other contextual factors including practice size, levels of deprivation, and geographical location are the main inhibitory factors of effectively implementing electronic consultations. As a result, the majority of included studies reported an increase in clinician and admin time, with many GPs reporting a duplication in workload, as opposed to the desired reduction. Findings from our review also identified a number of factors that could help facilitate the effective implementation of electronic consultations. These primarily focused on the provision of staff training, protocols, strategies, and guidance; enhanced patient awareness and education; strategic marketing; notification alerts, and patient and public involvement in the innovation, implementation, and evaluation stages.

### Comparison With Existing Literature

Similar to existing research, the included articles reported a shortcoming of electronic consultations in their current form [4], with expressed skepticism regarding their financial investment [3]. Furthermore, many health care professionals expressed concern that electronic consultation duplicated administrative and clinical workloads. Such findings have been widely reported by a number of studies [1,3,4,6,16]. However, it is important to note that this may be due to the absence of supporting factors identified in this review and the relatively new emergence of electronic consultations. Further exploration of whether the presence of facilitating factors improves electronic consultation would be beneficial. Finally, we found a limited amount of research exploring electronic consultation experiences and impact from a patient perspective [3,6,11], identifying a further area for future research.

### Strengths and Limitations

Strengths of this review include its exploration of both peer-reviewed and grey literature and novel application of a theoretical framework in the context of general practice and electronic consultations. However, its limitations must also be acknowledged. In line with scoping review practice, included articles were not quality appraised. The exclusion of non–English language texts may also have introduced research bias. Future research may benefit from a wider range of bibliographic databases, including technical databases such as IEEE. Exploration of any differences between private and free at the point of access service implementations/commissioning would also be beneficial*.*

### Implications for Practice

With these in mind, the implications from this review are clear. First, the implementation of electronic consultations appears most effective when both patients and staff members are involved in the design, implementation, and evaluation of their processes and outcomes. Second, the rationale, purpose, and intended use of electronic consultations needs to be effectively communicated to both patients and staff members to ensure appropriate use and implementation. This could be best achieved through targeted marketing as well as meaningful patient involvement to facilitate patient understanding and acceptability. Third, marketing materials should reflect the reality of the product proposed to effectively manage people’s expectations (ie, electronic consultation may not reduce workload to the extent originally promised). Efforts should also be made wherever possible to incorporate the factors identified as beneficial in electronic consultation implementation to achieve maximum success and impact. Finally, further work is required to explore the potential inequalities in electronic consultations, with evidence to suggest some patient groups may be disadvantaged or unintentionally excluded from electronic consultation opportunities [8,23]. Further exploration of the economic costs and benefits of electronic consultations from the perspective of patients, professionals, and commissioners would also be beneficial in informing current debates.

### Conclusion

In conclusion, the implementation of electronic consultations in primary care can be facilitated by the development of protocols and strategies, patient and staff education, accurate and targeted education, and meaningful patient and public involvement. Efforts should be made wherever possible to incorporate factors identified as beneficial in facilitating electronic consultations to ensure maximum impact and success. Further research exploring the economic impacts of electronic consultations would be beneficial from a patient, professional, and commissioner perspective.

## Abbreviations

GP: general practitioner
IT: information technology
NPT: Normalization Process Theory
